# Hydrolyzed Collagen from Salmon Skin Mitigates L-NAME-Induced Hypertension in Rats by Attenuating Oxidative Stress and Inflammation and Improving Vascular Remodeling

**DOI:** 10.3390/ijms27062805

**Published:** 2026-03-19

**Authors:** Pimchanok Mungmuang, Jiraporn Tocharus, Luckika Panthiya, Rattapong Sungnoon, Krisana Nilsuwan, Soottawat Benjakul, Chainarong Tocharus

**Affiliations:** 1 Department of Anatomy, Faculty of Medicine, Chiang Mai University, Chiang Mai 50200, Thailand; pimchanok_m29@hotmail.com; 2 Department of Physiology, Faculty of Medicine, Chiang Mai University, Chiang Mai 50200, Thailand; jiraporn.tocharus@cmu.ac.th (J.T.); sungnoon@gmail.com (R.S.); 3 Department of Radiological Technology, Faculty of Allied Health Sciences, Thammasat University, Pathum Thani 12120, Thailand; luckika_pa@hotmail.com; 4 International Center of Excellence in Seafood Science and Innovation, Faculty of Agro-Industry, Prince of Songkla University, Songkhla 90110, Thailand; krisana.n@psu.ac.th (K.N.); soottawat@psu.ac.th (S.B.); 5 Functional Food Research Center for Well-Being, Chiang Mai University, Chiang Mai 50200, Thailand

**Keywords:** hydrolyzed collagen, hypertension, nitric oxide, vascular dysfunction, inflammation

## Abstract

This study aimed to investigate whether hydrolyzed collagen (HC) derived from salmon skin could attenuate blood pressure and vascular damage in Nω-nitro-L-arginine methyl ester hydrochloride (L-NAME)-induced hypertensive rats. Hypertension was induced in rats by the oral administration of L-NAME (40 mg/kg/day) for eight weeks, while HC in two doses (50, 450 mg/kg) or enalapril at 10 mg/kg dissolved in water were concurrently administered via oral gavage for the last four weeks. Treatment with HC (450 mg/kg) and enalapril resulted in a reduction in systolic blood pressure, oxidative stress markers, and inflammatory cytokines. Production of serum nitric oxide (NO) was also increased, contributing to better aortic function. Histopathological analysis confirmed these changes, showing enhanced progression in the aorta structure. Vascular function was improved, as evidenced by significantly higher relaxation responses to acetylcholine (ACh) in the presence or absence of endothelium. These findings indicate that HC effectively lowered the blood pressure in hypertensive rats, potentially through mechanisms involving the modulation of oxidative stress and the expression of nitric oxide, reducing vascular inflammation and remodeling, hence enhancing vascular function.

## 1. Introduction

Hypertension is a common symptom of cardiovascular disease, characterized by high blood pressure. High blood pressure is also a major cause of cardiovascular disease such as ischemic heart disease, heart failure, stroke, atherosclerosis, and renal failure [1,2,3]. In blood vessels, the endothelium is a monolayer of cells that maintains vascular homeostasis and regulates vasomotor function, balancing vasodilation and vasoconstriction. Nitric oxide (NO), a vasodilator, is generated from L-arginine through the action of nitric oxide synthases (NOSs), which activate protective molecules against vascular damage, a hallmark of endothelial dysfunction [4]. The pathophysiology of cardiovascular diseases involves oxidative stress, as evidenced by the overproduction of reactive oxygen species (ROS) [5,6]. ROS upregulates the expression of the NADPH oxidase complex [7], the primary source of the superoxide anion (O_2_^•−^) in the vasculature [8]. Moreover, O_2_^•−^ interacts with NO to form reactive peroxynitrite (ONOO^−^), which uncouples NOS and reduces the vasodilatory effect of NO. The interaction between ROS and NO contributes to the downregulation of endothelial nitric oxide synthase (eNOS), a direct cause of endothelial dysfunction [9]. This process promotes vascular inflammation and remodeling, which play major roles in the pathophysiology of hypertension-related vascular damage. Additionally, the activation of nuclear factor kappa B (NF-κB) induces the expression of pro-atherogenic cytokines, such as tumor necrosis factor-α (TNF-α), and also adhesion molecules, for example, vascular cell adhesion molecule 1 (VCAM-1) and intercellular adhesion molecule 1 (ICAM-1) in the vascular endothelium. These molecules stimulate the further production of cytokines [10]. Matrix metalloproteinases (MMPs) are involved in vascular dysfunction and remodeling, with ROS stimulating the activation of MMP-2 and MMP-9. These enzymes contribute to the degradation of extracellular matrix (ECM) proteins in blood vessels and vascular smooth muscle cells, promoting the migration and proliferation of biomolecules within these cells [11,12]. Transforming growth factor-1 (TGF-1) plays a role in cell-to-cell signaling and collagen synthesis in vascular fibrosis [13,14]. These processes increase the efficiency of the action of endothelial nitric oxide synthase-derived nitric oxide on the activation of endothelial progenitor cells, thus enhancing the potential function. Chronic arterial hypertension led to Nω-nitro-L-arginine methyl ester hydrochloride (L-NAME) consumption and promoted the inhibition of NOS in induced hypertensive rats, resulting in NO-deficiency in the aortic cells. The increase in ROS decreased NO levels associated with arterial structural changes [15].

Previous studies have reported that hydrolyzed collagen (HC) from salmon skin has been reported to contain low molecular-weight bioactive peptides that show angiotensin-converting enzyme (ACE) inhibitory activity [16], where ACE plays a role in the renin–angiotensin–aldosterone system (RAAS) conducts to blood pressure regulation for promoting Ang I to Ang II mechanisms. Consequently, the effect of Ang II binding with the AT1 receptor leads to vasoconstriction. Moreover, these mechanisms increase in vascular tone [17,18]. In addition, it has the potential to significantly reduce systolic blood pressure as a consequence of salmon skin peptide intake [19]. Salmon skin collagen is a rich source of active peptides, making it valuable in the functional food industry. Collagen peptides have a triple-helix structure, mostly consisting of three α-chains. These polypeptide chains are predominantly composed of glycine and proline amino acids. The length is characterized by a repetitive sequence of Gly-X-Y amino acids, which enables the formation of hydrogen bonds within its molecules. Collagen denaturation is assessed by using enzymatic hydrolysis to produce hydrolyzed collagen, a functional protein whose properties are determined by amino acid composition, size, and shape [20,21,22]. HC from salmon skin consists of small peptides with low molecular weight (3–6 kDa). It consists of amino acid content for the enrichment in protein, which has stable digestion capabilities and is highly efficient in terms of bioactivity and pharmacological effects. The previous findings indicate that Atlantic salmon skin possesses antihypertensive activity and high digestibility due to its fragmented protein. Furthermore, it is linked to the prevention of atherosclerotic lesions on the aorta, which is associated with anti-inflammatory, antiplatelet, and antioxidative properties that contribute to the regulation of endothelial function [23]. Furthermore, fish protein hydrolysates are suggested to process peptides with high potential for antioxidant activity through free radical scavenging and antihypertensive activity to ACE-inhibition [24,25]. This study aimed to investigate the potential of HC containing bioactive peptides in providing arterial antihypertensive effects. These effects were mediated through antioxidant and anti-inflammatory properties and vascular remodeling for vascular protection in L-NAME-induced hypertensive rats.

## 2. Results

### 2.1. Effect of HC on SBP Changes in Rats Treated with L-NAME

**Figure 1:**
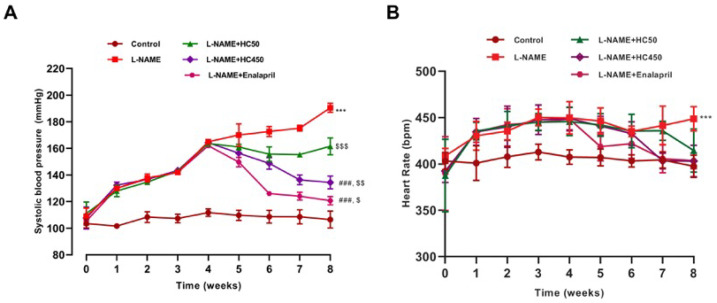
Effects of HC in rats treated with L-NAME. (**A**) Systolic blood pressure and (**B**) heart rate measurement period of treatments in all experimental groups. Data are expressed as mean ± S.E.M. One-way ANOVA followed by post hoc Dunnett’s multiple comparisons test. Statistical significance shown as *** *p* < 0.001 vs. control group, ^###^ *p* < 0.001 vs. L-NAME group, ^$^ *p* < 0.05, ^$$^ *p* < 0.01, ^$$$^ *p* < 0.001 vs. control group (*n* = 6 per group).

As shown in Figure 1A, the chronic administration of L-NAME induced a progressive and significant increase in SBP in rats (*p* < 0.001). Concomitant treatment with HC at a dose of 450 mg/kg significantly reduced the rise in blood pressure caused by L-NAME. HC at the dose of 450 mg/kg provided greater prevention against the increase in blood pressure than HC at 50 mg/kg (*p* < 0.001). Enalapril, used as the positive drug, inhibited the development of hypertension in L-NAME-treated rats (*p* < 0.001). However, blood pressure in the HC (50, 450 mg/kg) or enalapril-treated rats remained significantly higher than that in the control group (*p* < 0.05, 0.01, 0.001, respectively). Additionally, treatment with HC prevented L-NAME-induced tachycardia, as indicated by the heart rate being similar to that of the controls and the enalapril-treated rats (Figure 1B). The results suggest that HC exhibits an antihypertensive effect.

### 2.2. Improvement of Vascular Function in L-NAME Hypertensive Rats by HC

**Figure 2:**
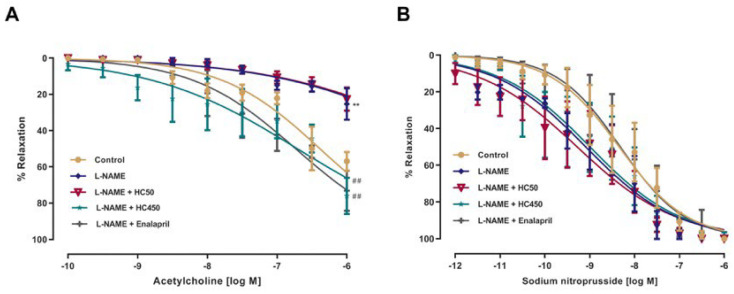
Effect of HC on vasorelaxation induced by (**A**) acetylcholine and (**B**) sodium nitroprusside in aortic rings pre-contracted with phenylephrine (10 μM). Data are expressed as mean ± S.E.M. One-way ANOVA followed by post hoc Dunnett’s multiple comparisons test. Statistical significance in value, ** *p* < 0.01 vs. control group, ^##^ *p* < 0.01 vs. L-NAME group (*n* = 6 per group).

The vascular reactivity to vasoactive agents was evaluated using the organ bath method. Isolated intact aortic rings from L-NAME-induced hypertensive rats were pretreated with phenylephrine (PE), followed by the cumulative addition of ACh (10^−10^–10^−5^ M) or SNP (10^−12^–10^−5^ M). The vasorelaxation responses to ACh were significantly impaired in aortic rings of L-NAME hypertensive rats compared with the control rats, as indicated by the lower Emax values (24.97 ± 5.74%), than the 98.72 ± 0.98% found in the control (*p* < 0.01). Treatment with HC at the dose of 450 mg/kg or enalapril significantly increased the vascular response to ACh compared with the L-NAME group, with Emax values of 82.89 ± 7.87% and 87.25 ± 7.56%, respectively (Figure 2A). However, at the dose of 50 mg/kg, HC did not result in a significant difference in vascular response to ACh compared with the L-NAME group (Emax 35.10 ± 5.78%). The vasorelaxation response to sodium nitroprusside did not differ significantly among all groups, as evidenced by no significant difference in the Emax values of nitroprusside-elicited relaxation (Figure 2B). These results suggest that HC might improve vascular dysfunction in L-NAME-induced hypertensive rats.

### 2.3. Attenuation of Plasma NO Levels and the Expression of eNOS in Aortic Tissues in L-NAME-Induced Hypertensive Rats by HC

**Figure 3:**
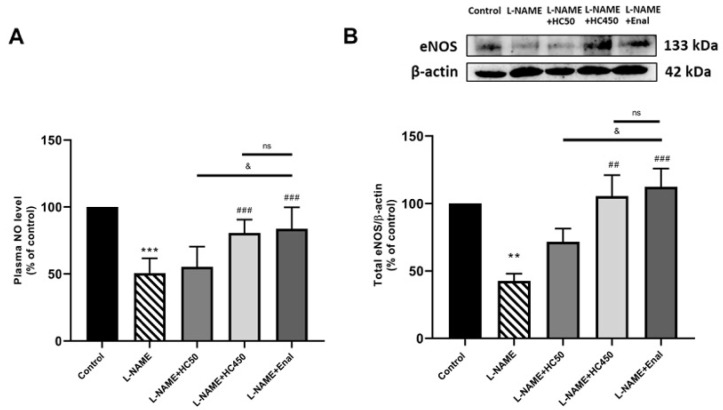
Effect of HC on the plasma level of (**A**) NO activity and (**B**) eNOS protein expression. Representative band analysis of eNOS protein expression. Data are expressed as mean ± S.E.M. One-way ANOVA followed by post hoc Dunnett’s multiple comparisons test. Statistical significance in value, ** *p* < 0.01, *** *p* < 0.001 vs. control group, ^##^ *p* < 0.01, ^###^ *p* < 0.001 vs. L-NAME group, ^&^ *p* < 0.05 vs. L-NAME + HC50, ns: non-significant (*n* = 6 per group).

Rats in the L-NAME- induced hypertensive group showed a significant reduction in plasma NO levels (Figure 3A) and the expression of eNOS (Figure 3B) in comparison with the control group. Treatment with HC at a dose of 450 mg/kg or enalapril resulted in significantly increased plasma NO levels and eNOS expression compared with the L-NAME-induced hypertensive control group. In contrast, HC (50 mg/kg) showed no significant effect.

### 2.4. Reduction in Oxidative Stress and the Inflammatory Response in L-NAME-Induced Hypertensive Rats by HC

**Figure 4:**
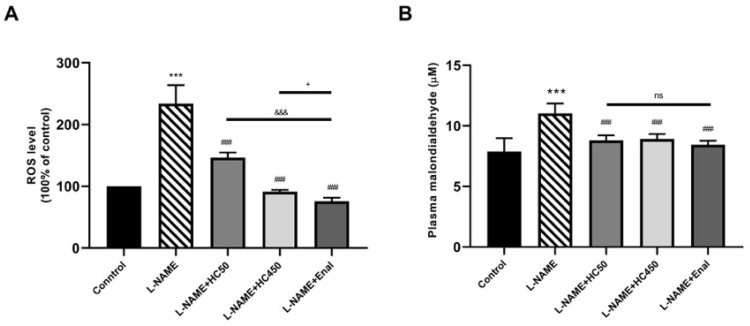
Effect of HC on oxidative stress. (**A**) ROS levels and (**B**) plasma MDA levels. Data are expressed as mean ± S.E.M. One-way ANOVA followed by post hoc Dunnett’s multiple comparisons test. Statistical significance in value, *** *p* < 0.001 vs. control group, ^###^ *p* < 0.001 vs. L-NAME group, ^&&&^ *p* < 0.001 vs. L-NAME + HC50, ^+^ *p* < 0.05 vs L-NAME + HC450, ns: non-significant (*n* = 6 per group).

**Figure 5:**
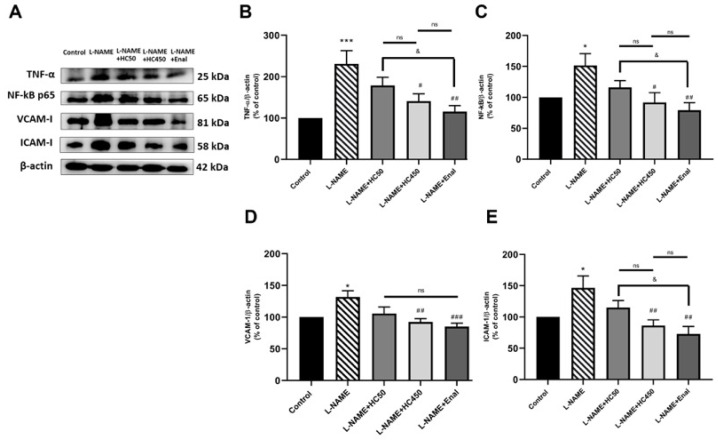
Effect of HC on the vascular inflammation in aortic tissues. (**A**) Representative bands of the TNF-α, NF-κB, VCAM-1, and ICAM-1 expression in aortic tissues, as investigated by Western blot analysis. The quantitative results of the expression of (**B**) TNF-α, (**C**) NF-κB, (**D**) VCAM-1, (**E**) ICAM-1 in each group. Data are expressed as mean ± S.E.M. One-way ANOVA followed by post hoc Dunnett’s multiple comparisons test. Statistical significance in value, * *p* < 0.05, *** *p* < 0.001 vs. control group, ^#^ *p* < 0.05, ^##^ *p* < 0.01, ^###^ *p* < 0.001 vs. L-NAME group, ^&^ *p* < 0.05 vs. L-NAME + HC50, ns: non-significant (*n* = 6 per group).

To further investigate the protective effects of HC in L-NAME-induced hypertensive rats, ROS levels in aortic tissues and plasma MDA levels were examined. As shown in Figure 4A,B, the ROS and plasma MDA levels were significantly higher (*p* < 0.001) in the L-NAME group compared with the control group. However, treatment with HC (50 and 450 mg/kg) and enalapril significantly reduced the ROS and MDA levels (*p* < 0.01). Moreover, HC (450 mg/kg) and enalapril produced a greater reduction in ROS than HC (50 mg/kg) (*p* < 0.001). Meanwhile, there was no significant difference in MDA levels among the HC (50 and 450 mg/kg) and enalapril groups. It has been reported that oxidative stress is closely linked to vascular inflammatory cascades. The results showed that the expressions of TNF-α, total NF-κB, VCAM1, and ICAM-1 were significantly increased in the L-NAME group (Figure 5A–E). However, treatment with HC at 450 mg/kg or enalapril significantly attenuated the expression of TNF-α, NF-κB, VCAM1, and ICAM-1 in comparison with the L-NAME-induced rats. There was no significant difference between the HC (450 mg/kg) and enalapril groups. These results indicate that HC has the potential to protect the vascular system by reducing oxidative stress and suppressing the production of inflammatory cytokines.

### 2.5. Attenuation of Vascular Remodeling in L-NAME-Induced Hypertensive Rats by HC

**Figure 6:**
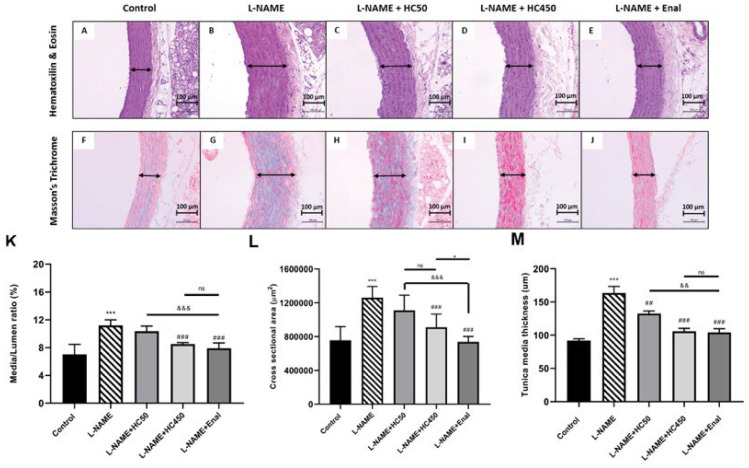
Effect of HC on histology and morphology of the aorta. Panels (**A**–**E**) show the histology of the aorta stained by hematoxylin and eosin staining (H&E). Panels (**F**–**J**) show Masson’s Trichrome staining for the collagen area. Represented in the normal control, L-NAME-induced hypertensive rat, L-NAME + HC (50 mg/kg), L-NAME + HC (450 mg/kg), and L-NAME + enalapril (10 mg/kg), to indicate the vascular medial thickening. Images were observed under a light microscope using 20× magnification. The scale bar represents 100 μm. Data show histological changes in vascular remodeling of the aorta. (**K**) Media/lumen ratio, (**L**) cross-sectional area, (**M**) tunica media thickness. Data are expressed as mean ± S.E.M. One-way ANOVA followed by post hoc Dunnett’s multiple comparisons test. Statistical significance in value, *** *p* < 0.001 vs. control group, ^##^ *p* < 0.01, ^###^ *p* < 0.001 vs. L-NAME group, ^&&^ *p* < 0.05, ^&&&^ *p* < 0.001, vs. L-NAME + HC50, ^+^ *p* < 0.05 vs. L-NAME + HC450, ns: non-significant (*n* = 6 per group).

**Figure 7:**
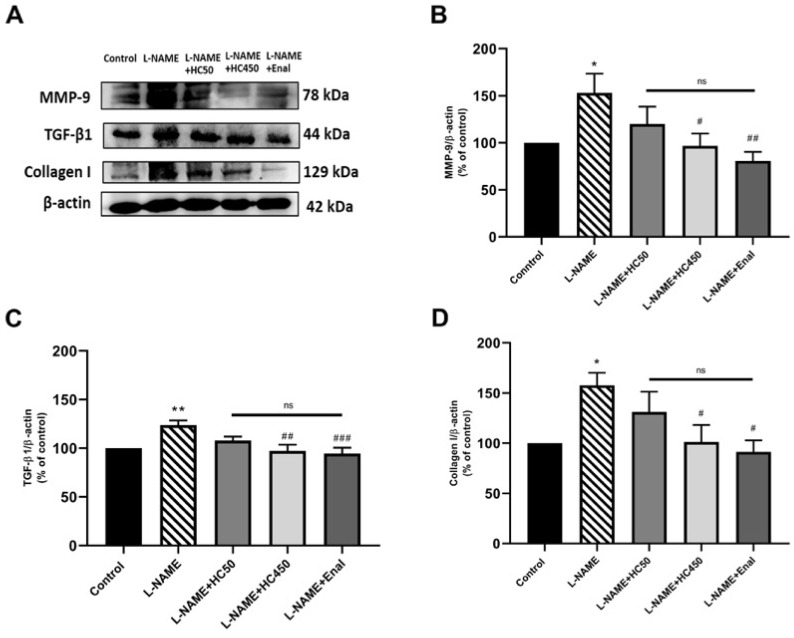
Effect of HC on the vascular remodeling in aortic tissues. (**A**) Representative bands of MMP-9, TGF-β I, and collagen I expression in aortic tissues, as investigated by Western blot analysis. The quantitative results of the expression of (**B**) MMP-9, (**C**) TGF-β I, and (**D**) collagen I. Representative band analysis of all protein expression. Data are expressed as mean ± S.E.M. One-way ANOVA followed by post hoc Dunnett’s multiple comparisons test, and differences between the two groups were determined by using Student’s *t*-test. Statistical significance in value, * *p* < 0.05, ** *p* < 0.01 vs. control group, ^#^ *p* < 0.05, ^##^ *p* < 0.01, ^###^ *p* < 0.001 vs. L-NAME group, ns = no significant differences among groups (*n* = 6 per group).

Histological analysis of the aorta in the control rats showed normal aortic layers including the tunica intima, media, and adventitia (Figure 6A). In contrast, the aortas of the L-NAME-induced rats exhibited progressive thickening of the tunica media structure (Figure 6B). Treatment with HC at doses of 50 mg/kg, 450 mg/kg, and enalapril resulted in a significant reduction in tunica media thickness in comparison with the L-NAME group (Figure 6M). Vascular remodeling in the L-NAME group was associated with a significant increase in the media/lumen ratio (Figure 6K) and cross-sectional area (Figure 6L) in comparison with the control group. However, HC treatment at doses of 450 mg/kg and enalapril significantly ameliorated these changes. Next, collagen deposition in the aortic smooth muscle was assessed using Masson’s trichrome staining. The aortas of L-NAME-induced hypertensive rats exhibited fibrosis in the tunica media and tunica adventitia compared with the control group (Figure 6G). This fibrosis was significantly reduced in the HC- and enalapril-treated groups (Figure 6I,J). To further investigate the effect of HC on vascular fibrosis in L-NAME-induced hypertensive rats, the expression levels of MMP-9, TGF-β1, and collagen 1 were analyzed by Western blotting. The results showed that L-NAME-induced rats had significantly increased expression of MMP-9, TGF-β1, and collagen 1 (Figure 7B–D). However, treatment with HC (450 mg/kg) or enalapril significantly reduced the expression level of these markers compared with the L-NAME group. These findings indicate that HC exerts a significant protective effect against hypertensive-induced vascular fibrosis.

## 3. Discussion

Based on the present study, HC significantly reduced blood pressure in L-NAME-induced hypertensive rats by attenuating oxidative stress, vascular inflammation, and vascular remodeling.

Previous studies have demonstrated that L-NAME, an NOS inhibitor, induces hypertension through mechanisms involving vascular dysfunction, inflammation, remodeling, and fibrosis [26,27]. Consistent with these studies, rats treated with L-NAME also exhibited increased SBP and heart rate [28]. Vascular dysfunction is a key contributor to hypertension [29]. The present study demonstrated that HC reduced blood pressure by improving vascular function. Previous studies have shown that L-NAME induces vascular dysfunction in hypertensive rats, and our results are consistent with these findings, as vascular dysfunction was also observed in the L-NAME-treated rats [29]. Our data further suggest that HC preserved endothelial function, as indicated by enhanced vasorelaxation in response to acetylcholine (ACh), which stimulates endothelial cells to increase NO bioavailability and thereby promotes vascular relaxation. In contrast, HC did not significantly affect responses to sodium nitroprusside (SNP), an NO donor that regulates vascular function by activating the cGMP signaling pathway and reducing Ca^2+^ levels in vascular smooth muscle cells [30].

Chronic L-NAME administration prevented NO generation and accelerated O_2_^•−^ production from NADPH oxidase, leading to ONOO- formation [31]. The increase in ROS contributes to the overexpression of plasma MDA levels, a by-product of lipid peroxidation and a biomarker of oxidative stress. This oxidative stress is linked to the activation of the expression of pro-inflammatory cytokines and various downstream inflammatory signaling pathways such as NF-κB [32]. NO is primarily produced in blood vessels by endothelial cells through the conversion of L-arginine to NO, catalyzed by eNOS, which plays a crucial role in the maintenance of endothelial homeostasis [33]. NO is essential for the activation of cGMP and its downstream PKG signaling pathway, which inhibits Ca^2+^ influx and promotes vasorelaxation [34]. These mechanisms are directly involved in the development of hypertension and vascular dysfunction. The results suggest that HC attenuates oxidative stress by reducing ROS and MDA accumulation, while simultaneously enhancing NO production and upregulating eNOS protein expression. This upregulation may promote eNOS–calmodulin binding, thereby increasing eNOS activity. eNOS can also be activated by agonists such as bradykinin and acetylcholine through G protein-dependent signaling pathways that may regulate eNOS activity [35]. In a previous study, buthionine sulfoximine (BSO) was used to induce oxidative stress, and both in vitro and in vivo experiments examined the effects of NO on eNOS expression [36]. In this study, the HC groups also exhibited anti-hypertensive effects due to NO production, consistent with previous studies cited above. Oxidative stress-induced cell damage contributes to vascular inflammation, as indicated by increased TNF-α expression, NF-κB activation, and elevated VCAM-1 and ICAM-1 levels in the aorta. TNF-α activates NF-κB, a crucial factor in inflammatory processes. Within the vascular wall, NF-κB activation induces the expression of VCAM-1, facilitating monocyte adhesion to endothelial cells [37,38]. Upregulation of VCAM-1 and ICAM-1 promotes lymphocyte migration, contributing to endothelial dysfunction and pathological inflammation [39]. The present study found that HC treatment reduced vascular inflammation, as evidenced by a decrease in the expression of TNF-α, NF-κB, VCAM-1, and ICAM-1, ultimately leading to a reduction in inflammatory factors in aortic tissues, where, according to previous studies, the expression of inflammatory cytokines is decreased. Prolonged inflammation promotes vascular remodeling and fibrosis by increasing the expression of TGF-β1, which interacts with vascular smooth muscle cells, stimulating growth factors and proliferative activity. This process results in the accumulation of collagen, degradation of elastin, and extracellular matrix (ECM) remodeling, resulting in tissue fibrosis. MMP-9, a gelatinase involved in basement membrane degradation, further contributes to fibrosis formation [40]. Additionally, the activation of MMP-9 stimulates TGF-β1 signaling, inducing the expression of collagen-1 and fibronectin accumulation. These phenomena lead to arterial stiffness and elevated blood pressure [41,42]. This study demonstrates that HC reduces the expression of TGF-β1, MMP-9, and collagen-1 in aortic tissues by modulating MMPs and TGF-β1-mediated vascular remodeling, thereby decreasing the deposition of collagen. Histological analysis was consistent with the Western blot results, revealing that excessive collagen synthesis occurred in the L-NAME-treated rats [43]. The HC treatment demonstrated the ability to prevent vascular injury, as the decrease in these cytokines contributed to reversing vascular change, as previously referred to in the study. However, the research on the effect of HC on vascular histology change in the context of hypertension remains limited. Consequently, HC treatment was investigated in this study. In L-NAME-hypertensive rats, H&E staining showed thickened vascular walls, while Masson’s trichrome staining demonstrated abundant collagen deposition in the tunica media, correlating with increased M/L and MT ratios and CSA density. However, treatment with HC at high concentrations significantly reduced the collagen volume density, suggesting its potential to improve vascular remodeling and exert an anti-hypertensive effect. According to the hypertensive rat, the histology results also indicated the capacity of vascular integrity, similar to a previous study [44]. These results support the hypothesis that HC treatment can improve vascular function and reduce several cytokines, thereby contributing to the overall antihypertensive mechanisms.

The properties of hydrolyzed collagen peptides have bioactive compounds with functional effects [45,46,47]. As collagen plays a protective role in humans, it is often provided in the form of dietary supplements [48]. The hydrolyzed collagen can be utilized in a variety of food product industries, as it is a product obtained through hydrolysis to achieve enhanced quality. This study demonstrated that high doses of HC (450 mg/kg/day) were highly effective and showed significant results, including potent inhibitory effects against hypertension in rats. Based on previous studies, HC was investigated across varying doses, and it was found to be non-toxic following oral administration in rats [49,50,51]. Meanwhile, no critical adverse reactions were observed during HC treatment in this study. These findings suggest that the use of HC is safe at the quantities administered.

HC was shown to be as effective as enalapril in protecting vascular function by reducing blood pressure, combating oxidative stress, alleviating vascular inflammation, and preventing vascular remodeling. Therefore, HC could function as a bioactive food supplement with potential anti-hypertensive properties. Its therapeutic potential may be beneficial for the prevention of vascular damage associated with hypertension.

## 4. Materials and Methods

### 4.1. Preparation of Hydrolyzed Collagen from Salmon Skin

Frozen skins of sockeye salmon (*Oncorhynchus nerka*) were reduced to small pieces (3.0 × 3.0 cm^2^), and the removal of non-collagenous protein was conducted using an alkaline solution as detailed by the method in [52]. Thereafter, the prepared skin was subjected to a swelling process using 0.05 M citric acid (1:10, *w*/*v*) with gentle agitation for 15 min and left to stand for 45 min. The swollen skins were rinsed with water until a neutral pH was obtained.

Hydrolyzed collagen (HC) was produced following the method in [53]. The swollen skins were mixed with distilled water (1:5, *w*/*v*) and adjusted to pH 8. Papain and alcalase were added at 3% and 4% (*w*/*w*, based on solid content), respectively, in the prepared mixture. Hydrolysis was carried out at 60 °C with continuous stirring for 4 h. Finally, the enzymes in the hydrolysate were inactivated at 90 °C for 15 min.

The HC solution was further defatted with the aid of a disk stack centrifugal separator (SPX FLOW Technology Italia S.p.A., Milan, Italy). The feed rate of 2.0 L/min with nine cycles was used. HC solution was collected and lyophilized [53]. The lyophilized HC sample was further defatted using isopropanol for three cycles. HC powder was packed in nylon polyethylene laminated bags and stored at −20 °C. The degree of hydrolysis was 35%, and the peptide with an MW of 1460 Da was predominant, followed by those with an MW of 1975 and 1530 Da, respectively, as determined by the methods in [54,55].

### 4.2. Animals

**Figure 8:**
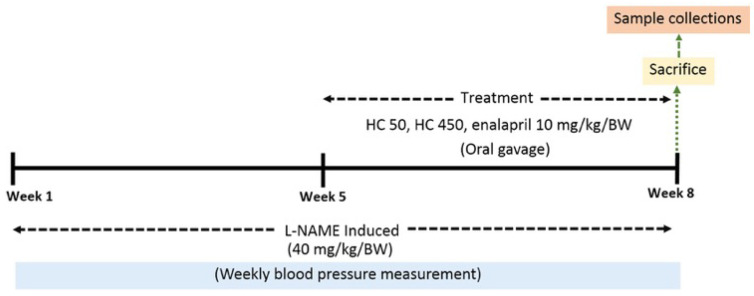
Experimental protocol for L-NAME-induced hypertension in rats over 8 weeks, with treatment using HC (50 or 450 mg/kg) or enalapril during the final 4 weeks.

Male Wistar rats (weighing 200–220 g) were obtained from Nomura Siam International, Bangkok, Thailand. The rats were fed on a standard diet and housed in a room with controlled environmental conditions, including a temperature of 24 ± 1 °C and a 12-h light–dark cycle. The animal care and experimental processes in this study were approved by the Institutional Animal Care and Use Committee at the Faculty of Medicine, Chiang Mai University, Thailand (protocol number 08/2565) and were performed in accordance with the National Institute of Health’s Guide for the Care and Use of Laboratory Animals. Following a 1-week acclimatization period, the rats were randomly divided into five groups (n = 6 per group) including: Group 1—Normal + vehicle (Distilled water); Group 2—L-NAME + vehicle; Group 3—L-NAME + HC (50 mg/kg/day); Group 4—L-NAME + HC (450 mg/kg/day); and Group 5—L-NAME + enalapril (10 mg/kg/day, Berlin Pharmaceutical Industry, Thailand). The L-NAME rats were treated with L-NAME (40 mg/kg/day, Sigma-Aldrich, St. Louis, MO, USA). L-NAME was added to their drinking water for eight weeks to induce hypertension. HC and enalapril were dissolved in water and administered via oral gavage daily for the last 4 weeks (Figure 8). Enalapril, an angiotensin-converting enzyme (ACE) inhibitor, served as the positive control, consistent with previous studies [56,57]. Enalapril has been shown to reduce blood pressure and oxidative stress markers, improve endothelial function, and restore aortic eNOS expression [56,57]. The beneficial effects of enalapril in hypertension are attributable not only to ACE inhibition, but also to its potent antioxidant activity. At the end of the experiment, the rats were euthanized with isoflurane, and the aorta and blood were collected and stored at −80 °C.

### 4.3. Blood Pressure Measurement

The non-invasive tail-cuff method (MK-1030, MUROMACHI KIKAI Co., Ltd., Tokyo, Japan) was used for the measurement of systolic blood pressure (SBP), along with heart rate measurements. Rats were held in a heating box, allowing for blood pressure measurement (PMB-1030 Preheat & Measuring Box, Tokyo, Japan). The SBP was recorded each week until the end of the treatment period.

### 4.4. Vascular Reactivity Study

The thoracic aortas were quickly isolated and incubated in Krebs solution (composition in mM: NaCl 122, KCl 4.9, HEPES 10, KH_2_PO_4_ 0.5, NaH_2_PO4 0.5, MgCl_2_ 1.0, glucose 11.0, and CaCl_2_ 18 and pH 7.3) in an organ bath system at 37 °C under 95% O_2_–5% CO_2_. The connective tissues were carefully removed and cut into rings that were 3 mm in length. The aortic rings were fixed with hooks by connecting to an isometric force transducer (Iworx System, Inc., Dover, NH 03820, USA). Tone was recorded in each vessel using LabChart 7 (ADInstruments, Sydney, Australia). The resting tension rings were 1 g, with an equilibration period of 60 min. After normalization, the vessels were pre-contracted with phenylephrine (PE; 1 µM) and exposed to increasing concentrations of acetylcholine (ACh, Sigma Aldrich, USA) or sodium nitroprusside (SNP, Sigma Aldrich, USA) to test vascular relaxation.

### 4.5. Preparation of the Aorta for Biochemical Assay

Aortic tissues were homogenized in RIPA lysis buffer (150 mM NaCl, 50 mM Tris-HCl; pH 8.0, 1% NP-40, 0.5% sodium deoxycholate, 0.1% SDS, and 5 mM EDTA; pH 8.0) with proteinase inhibitor. Each aorta was filled with liquid nitrogen. The homogenate was centrifuged at 12,000 rpm for 15 min at 4 °C. The supernatant was collected and stored at −80 °C for further analysis.

### 4.6. Measurement of Plasma NO Levels

The plasma NO level was measured by the Griess reagent method [58]. Briefly, the supernatants were incubated with 1% sulfanilamide in 5% phosphoric acid and 0.1% N-(1-Naphthyl) ethylenediamine-HCl for 10 min. The absorbance was detected using a microplate reader at a wavelength of 540 nm (BioTek Instruments, Winooski, VT, USA).

### 4.7. Measurement of ROS Production

The aortic homogenates were assessed for ROS production by using a ROS-sensitive dye, 2′,7′-dichlorofluorescein diacetate (H_2_DCFDA). The supernatant was incubated with 20 µM H_2_DCFDA working solution and then incubated for 30 min at 37 °C in the dark. The level of DCF fluorescence was measured using a fluorescent microplate reader (BioTek Instruments, Winooski, VT, USA) with an excitation wavelength of 485 nm and an emission wavelength of 538 nm.

### 4.8. Measurement of Lipid Peroxidation

The malondialdehyde (MDA) level was used to evaluate lipid peroxidation in the aortic tissue homogenate [59]. The tissue homogenate was incubated with 10% trichloroacetic acid (TCA) and 0.67% (*w*/*v*) thiobarbituric acid and heated at 100 °C for 30 min. The supernatant was measured at the wavelength of 532 nm in a microplate reader (BioTek Instruments, Winooski, VT, USA).

### 4.9. Western Blot Analysis

The total protein in aortic tissue homogenates was determined using the Bradford protein assay (Bio-Rad, Hercules, CA, USA) with bovine serum albumin as a standard. The equivalent quantities of protein samples were separated by using sodium dodecyl sulfate-polyacrylamide gel electrophoresis (SDS-PAGE) using a 10–12% gel. After being separated, proteins were transferred to a polyvinylidene difluoride (PVDF) membrane. The membrane was blocked with 5% skimmed milk in Tris-buffered saline (TBS) containing 0.1% Tween 20 (TBS-T) for 2 h at room temperature. Then, membranes were incubated overnight at 4 °C with their primary antibody. The following antibodies were used: anti-eNOS (1:1000; Abcam, Cambridge, UK), anti-TNF-α (1:1000; Abcam, Cambridge, UK), anti-NF-κB (1:1000; Cell Signaling, Danvers, MA, USA), anti-VCAM1 (1:1000; Cell Signaling, Danvers, MA, USA), anti-ICAM-1 (1:1000; Abcam, Cambridge, UK), anti-MMP-9 (1:1000; Merck, Darmstadt, Germany), anti-TGF-β1 (1:1000; Affinity bioscience, Changzhou, China), and anti-collagen type I (1:1000; ThermoFisher Scientific, Waltham, MA, USA). After washing with TBS, the membrane was incubated with horseradish peroxidase-conjugated secondary antibody for 2 h at room temperature. The membranes were incubated with Immobilon Forte Western HRP substrate (Millipore, MI, USA), and the signals were detected by using a ChemiDoc™ with chemiluminescence system (Immobilon^TM^ Western, Millipore, Burlington, MA, USA). Relative intensities were calculated by using ImageJ software version 1.44, using β-actin as an internal control.

### 4.10. Histological Analysis

The aorta was fixed in 10% formalin for 24 h. The samples were embedded in paraffin, and the tissues were cut into sections (5 μm thick) using a microtome (Leica, Wetzlar, Germany). Visualization of the aorta was carried out via hematoxylin and eosin (H&E) staining for the tissue sections and Masson’s trichrome for collagen deposition, following the method described in an earlier study [60]. The aortic sections were examined using a light microscope (Olympus System Model BX51, Japan). The density of aortic change was calculated using the sectional area, while remodeling of the aorta was assessed via tunica media and wall thickness, ratio of media thickness (MT), cross-sectional area (CSA), and media/lumen (M/L). The average measurement was calculated from three sections for each group.

### 4.11. Statistical Analysis

All values are expressed as the mean ± S.E.M. Data of multiple group comparisons were analyzed by one-way analysis of variance (ANOVA) followed by post hoc Dunnett’s test using GraphPad Prism 8. Statistical significance was considered at a *p*-value of ≤ 0.05.

## 5. Conclusions

HC was shown to effectively protect vascular function by reducing blood pressure, combating oxidative stress, alleviating vascular inflammation, and preventing vascular remodeling. Therefore, HC could function as a bioactive food supplement with potential anti-hypertensive properties. Its therapeutic potential may be beneficial for the prevention of vascular damage associated with hypertension.

## Data Availability

The original contributions presented in this study are included in the article. Further inquiries can be directed to the corresponding author.

## Abbreviations

The following abbreviations are used in this manuscript:

HC	Hydrolyzed Collagen
L-NAME	Nω-Nitro-L-Arginine Methyl Ester
NO	Nitric Oxide
ACh	Acetylcholine
SNP	Nitroprusside
NOS	Nitric Oxide Synthase
ROS	Reactive Oxygen Species
NADPH	Nicotinamide Adenine Dinucleotide Phosphate
O_2_^•−^	Superoxide Anion
ONOO^−^	Peroxynitrite
eNOS	Endothelial Nitric Oxide Synthase
NF-κB	Nuclear Factor Kappa B
TNF-α	Tumor Necrosis Factor-α
VCAM-1	Vascular Cell Adhesion Molecule-1
ICAM-1	Intercellular Adhesion Molecule-1
MMP	Matrix Metalloproteinase
ACE	Angiotensin-Converting Enzyme
RAAS	Renin–Angiotensin–Aldosterone System
SBP	Systolic Blood Pressure
PE	Phenylephrine
MDA	Malondialdehyde
TGF-β1	Transforming Growth Factor-β1
cGMP	Cyclic Guanosine Monophosphate
Ca^2+^	Calcium Ion
SERCA	Sarcoendoplasmic Reticulum Calcium ATPase
PKG	Protein Kinase G
ECM	Extracellular matrix
M/L	Media/Lumen
CSA	Cross-Sectional Area
MT	Media Thickness
Emax	Maximum Effect
